# Uncertainty quantification via polynomial chaos expansion of myocardial fibre orientation and cardiac activation patterns

**DOI:** 10.1113/JP287746

**Published:** 2025-08-22

**Authors:** Lindsay C. R. Tanner, Anna Busatto, Jake A. Bergquist, Wilson W. Good, Brian Zenger, Gernot Plank, Akil Narayan, Karli Gillette, Rob S. MacLeod

**Affiliations:** ^1^ Scientific Computing and Imaging Institute University of Utah Salt Lake City Utah USA; ^2^ Nora Eccles Cardiovascular Research and Training Institute University of Utah Salt Lake City Utah USA; ^3^ Department of Biomedical Engineering University of Utah Salt Lake City Utah USA; ^4^ Washington University School of Medicine St. Louis Missouri USA; ^5^ Division of Biophysics and Medical Physics, Gottfried Schatz Research Center Medical University of Graz Graz Austria; ^6^ Department of Mathematics University of Utah Salt Lake City Utah USA

**Keywords:** biological simulations, cardiac electrophysiology, myocardial fibres, uncertainty quantification

## Abstract

**Abstract:**

Predictive models and computational simulations of cardiac electrophysiology depend on precise anatomical representations, including the local myocardial fibre structure. However, obtaining patient‐specific fibre information is challenging. In addition, the influence of physiological variability in fibre orientation on cardiac activation simulations is poorly understood. We implemented rule‐based algorithms to generate fibres and robust uncertainty quantification methods to determine model output variability with respect to ventricular activation sequences. We used polynomial chaos, which reduces computational demands by using an emulator to approximate the underlying forward model. Our study examined activation sequences in response to nine stimuli and five metrics quantifying essential features of the activation sequence. The results indicated that the primary fibre orientation impacts the overall spread of activation, which could impact more complex patterns of activation; however, there is minimal impact on the location of discrete activation features, such as breakthrough sites. For free wall stimuli, the standard deviation (STD) was highest near the stimulus site, diminishing with distance. Apical stimuli showed complementary STD patterns, with epicardial pacing maximizing STD in the right basal area and endocardial pacing in the left. Ventricular junction stimuli exhibited symmetrical STD patterns, low near the stimulus but increasing sharply towards the apex, peaking on the left in the apical region. Furthermore, variability in the imbrication or helix angle did not impact the activation sequences. We conclude that in many relevant modelling contexts, the variability in myocardial fibre orientation can play an important role in the resulting activation sequences and should be accounted for.

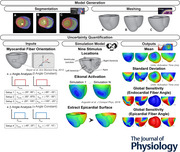

**Key points:**

- The primary fibre orientation has modest impact on activation duration and location of most discrete activation features for ectopic stimuli, but introduces variability in activation sequence.
- For free wall stimuli, the standard deviation (STD) was highest on the stimulated surface, indicating that deviations are largest in early activation and diminish as activation reaches remote heart regions.
- For apical stimuli, activation patterns were insensitive to fibre orientation variations, but the STD had complementary maxima, strongly dependent on pacing surface. Epicardial pacing produced largest STD in right basal area while endocardial pacing affected left basal area, indicating strong dependence on fibre structure.
- For stimuli at both anterior and posterior ventricular junctions, STD patterns were symmetrical, with low values near stimulus and sharp increases towards apex, peaking in left apical region.
- The helical fibre orientation showed no relevant fluctuations in activation sequence.

## Graphical abstract

**Abstract figure legend** Graphical representation of methods. We implemented three biventricular geometric models (Zenger et al., 2020) with rule‐based myocardial fibre orientations (Bayer et al., 2018). We evaluated variability in the fibre orientation via four sets of parameter distributions to determine the role of the primary and imbrication angles (Streeter et al., 1969; Lombaert et al., 2012). We applied polynomial chaos expansion using UncertainSCI (Narayan et al., 2023; Tate et al., 2023) to model parameter variability and its impact on epicardial activation times simulated in CARPentry using the eikonal model (Augustin et al., 2016). Statistical moments, such as mean, standard deviation (STD) and global sensitivities, were calculated for epicardial activation times.

## Introduction

The representation of myocardial fibre architecture is an essential element in computational models of excitation propagation in the heart that seek to predict behaviours under normal and pathophysiological conditions (Corral‐Acero et al., 2020; Plank et al., 2021; Gillette et al., 2021; Strocchi et al., 2023; Qian et al., 2023; Trayanova & Prakosa, 2024). Common approaches to capturing this architecture include image‐based measurements from individual hearts (Reese et al., 1995; Hsu & Henriquez, 2001) and rule‐based methods (Bayer et al., 2018). The measurements are based on diffusion‐tensor magnetic resonance imaging (DT‐MRI) from which it is possible to extract discrete tensor fields of fibre direction (Reese et al., 1995; Hsu & Henriquez, 2001). Despite substantial development, DT‐MRI remains limited by noisy, costly image acquisition and the need for explanted tissue, making patient‐specific application impractical. High‐quality images are difficult to obtain, even in controlled experimental or post‐mortem settings, and are rarely available in large, public databases, further restricting the utility of this imaging modality (Alexander ET AL., 2001; Rohmer et al., 2007). On the contrary, rule‐based algorithms are based on mathematical approximations of observed fibre structure that can be adjusted to any heart once individual gross anatomy is available (Bayer et al., 2018). While not perfect, rule‐based fibre directions are widely used and accepted within the research community as sufficiently accurate for many types of analyses (Gillette et al., 2021; Neic et al., 2020; Trayanova & Prakosa, 2024).

Whether obtained from image‐based measurements or rule‐based approaches, the impact of errors in myocardial fibre orientation on simulated propagation is not completely understood. Previous research has shown that the spread of excitation through the myocardium is influenced by both the tissue's electrical conductivity and the orientation of its fibres. These fibres reflect the organized arrangement of cardiac cells (myocytes) within the heart muscle (Ingels, 1997; Taccardi et al., 2008). The local orientations of these myocardial fibres vary smoothly throughout the heart, and their anisotropic shape, together with the gap junctions that connect them, cause preferential spread of excitation along their long axis. There is a biological variation in fibre orientation of up to 20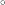 on the epicardial and endocardial surfaces (Lombaert et al., 2012), the effects of which remain largely unknown, either in terms of physiology or in computational modelling. While Muzikant et al. and Franzone et al. have described some effects of variable fibre orientation on simulated myocardial activation sequences, these studies lacked comprehensive statistical quantification (Muzikant et al., 2002; Franzone et al., 1998). On the contrary, Johnston et al. and Quaglino et al. have applied advanced statistical techniques to quantify the relationship between fibre orientation and myocardial activation; however, these studies lacked comprehensive evaluation over the full range of possible fibre orientations in a realistic heart model (Johnston et al., 2018; Quaglino et al., 2018). Johnston et al. implemented a slab model that lacked the full effect of the ventricles, whereas Quaglino et al. used realistic geometry but did not evaluate the activation sequence spatially (Johnston et al., 2018; Quaglino et al., 2018).

Statistical methods for quantifying the effects of uncertainty in the parameters that drive a simulation vary based on system complexity. Monte Carlo methods, widely used for uncertainty quantification, randomly sample the parameter space but often require millions of simulations, making them computationally prohibitive (Bullard & Sebald, 1988; Rubinstein & Kroese, 2016). Simplified alternatives, such as brute‐force methods and range‐finding experiments, reduce costs by undersampling or testing parameter extremes but rely on linearity assumptions that may not hold (Heule & Kullmann, 2017). More efficient techniques, like Gaussian process emulation (Bastos & O'Hagan, 2009) and polynomial chaos expansions (Xiu & Karniadakis, 2002), build surrogate models to approximate system responses with fewer simulations by leveraging mathematical assumptions about the underlying stochastic process (Narayan et al., 2023). Polynomial chaos expansion is particularly effective for systems with known distributions and manageable dimensions; researchers have shown its utility in areas like cardiac mechanics (Salvador et al., 2024; Berggren et al., 2024), cellular electrophysiology (Geneser et al., 2007; Clayton et al., 2020), myocardial propagation (Johnston et al., 2018) and bioelectricity (Geneser et al., 2008; Swenson et al., 2011; Bergquist et al., 2023).

The goal of this study is to quantify how variability in fibre orientation influences activation timing and spatial patterns. To explore the impact that errors in fibre orientation have on simulated propagation, we applied uncertainty quantification via polynomial chaos expansion to simulated cardiac activation from ventricularly paced beats. To simulate cardiac propagation efficiently, we used a model based on the eikonal approximation of the spread of activation (Vigmond et al., 2008; Gillette et al., 2021) along with rule‐based fibre orientations (Bayer et al., 2018), to which we applied robust uncertainty quantification algorithms (Narayan et al., 2023; Tate et al., 2023). The results included detailed maps of model sensitivity captured with robust statistical values. We carried out these studies on the three different hearts following pacing from nine anatomically varied sites across the ventricles of each heart and evaluated the effects on activation patterns over the epicardial surfaces. To capture and evaluate the global effects of fibre orientation, we also calculated five metrics quantifying key features of the epicardial activation sequence, based on previous mechanistic studies by Taccardi et al. (2008).

## Methods

**Figure 1:**
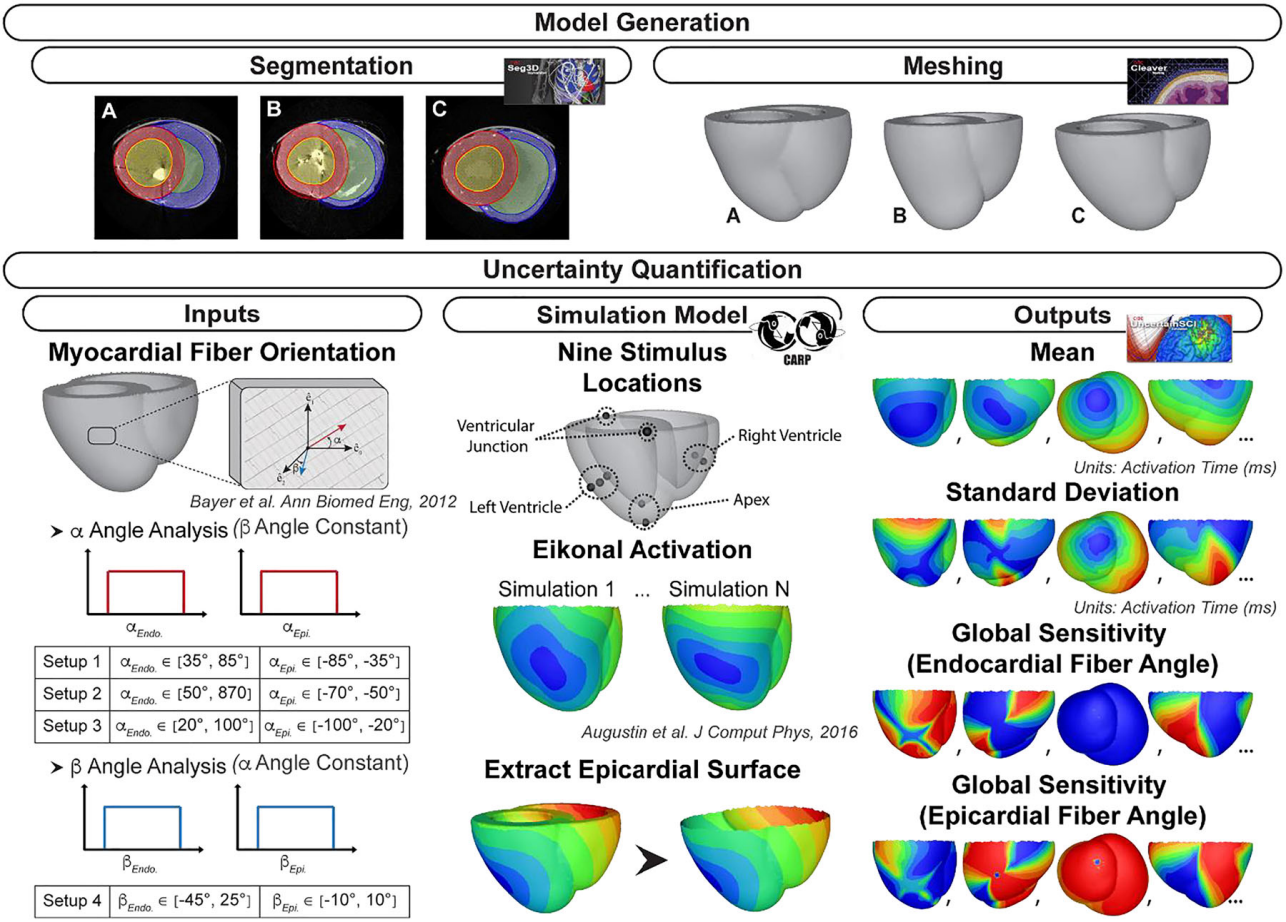
Graphical representation of methods We implemented three biventricular geometric models (Zenger et al., 2020) with rule‐based myocardial fibre orientations (Bayer et al., 2018). We evaluated variability in the fibre orientation via four sets of parameter distributions to determine the role of the primary and imbrication angles (Streeter et al., 1969; Lombaert et al., 2012). We applied polynomial chaos expansion using UncertainSCI (Narayan et al., 2023; Tate et al., 2023) to model parameter variability and its impact on epicardial activation times simulated in CARPentry using the eikonal model (Augustin et al., 2016). Statistical moments, such as mean, standard deviation (STD) and global sensitivities, were calculated for epicardial activation times.

Our methods are summarized in Fig. 1 and covered in more detail in a separate methods report (Tanner et al., 2025). Briefly, we created biventricular geometric models from scans of three explanted porcine hearts (Zenger et al., 2020) with rule‐based myocardial fibre orientations (Bayer et al., 2018). We evaluated the simulated spread of activation in the face of controlled variability in the orientation of the primary and helix angles defined by four sets of parameter distributions (Streeter et al., 1969; Lombaert et al., 2012). We applied polynomial chaos expansion using our custom open‐source software, UncertainSCI (Narayan et al., 2023; Tate et al., 2023), to model parameter variability and its impact on activation times simulated using the CARPentry software implementation of the eikonal model (Augustin et al., 2016). Statistical moments, such as mean, standard deviation (STD) and global sensitivities, were calculated and visualized for epicardial activation times. Furthermore, we quantified variability in the epicardial activation sequence using five additional metrics, described below.

### Geometric model generation

Three biventricular geometric models were generated from magnetic resonance images of explanted porcine hearts through segmentation using the open‐source software Seg3D (www.sci.utah.edu/software/seg3d). The hearts were similar in size, volumes of 69.3, 66.7 and 65.4 cm 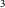, and were derived from high‐resolution MRI scans. Finite‐element meshes with a target mean edge length of 700 μ m were generated within another open‐source meshing software Cleaver (www.sci.utah.edu/software/cleaver). For more details on the model generation, please refer to Zenger et al. (2020). The models are referred to subsequently as Geometries A–C. The tissue acquisition was approved by the University of Utah IACUC (Protocol #20‐11001) following all institutional animal care and user guidelines.

### Myocardial fibre orientations

Myocardial fibre orientations were incorporated into each model using rule‐based fibre orientations (Bayer et al., 2018). Such algorithms generally assume a linear rotation of the fibre angle α and the helix angle β from the endocardium to the epicardium (the angles are defined in Fig. 1) (Bayer et al., 2018). We set the baseline or nominal epicardial and endocardial fibres from literature values (Streeter et al., 1969; Lombaert et al., 2012) to −60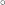 ($\alpha_{epi}$) and 60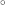 ($\alpha_{endo}$), respectively, and the helix epicardial and endocardial fibres to 0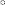 ($\beta_{epi}$) and −35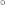 ($\beta_{endo}$), respectively. These values are presented in Table 1.

**Table 1:** Fibre orientations parameters for the α fibre angle on the epicardial ($\alpha_{epi}$) and endocardial ($\alpha_{endo}$) surfaces and β fibre angle on the epicardial ($\beta_{epi}$) and endocardial ($\beta_{endo}$) surfaces. The table includes baseline values and the ranges used for each of the four studies of the effect of fibre angle uncertainty on the activation sequence

	$\alpha_{epi}$	$\alpha_{endo}$	$\beta_{epi}$	$\beta_{endo}$
Baseline orientation	−60 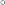	60 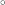	0 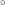	−35 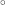
Physiological α angle range	−85 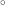 to −35 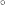	35 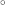 to 85 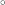	0 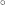	−35 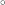
Narrow α angle range	−70 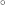 to −50 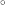	50 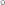 to 70 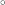	0 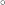	−35 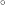
Broad α angle range	−100 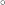 to −20 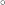	20 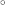 to 100 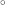	0 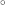	−35 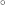
Physiological β angle range	−60 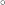	60 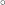	−10 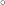 to 10 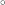	−45 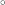 to −25 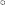

The variability of parameters in UQ analyses is best captured through probability distributions when such information is available, for example, from measurements. When such information is not robustly known, one can assign a uniform probability across a predetermined, hopefully reasonable, range of values. We used the latter approach to implement four individual sets of parameter distributions: (1) to evaluate the response to the α angle, we altered $\alpha_{epi}$ and $\alpha_{endo}$ uniformly between −85 to −35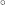 and 35 to 85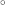, respectively, in what the literature suggests is a physiological range (Streeter et al., 1969; Lombaert et al., 2012). The β angle remained constant at the baseline value. We further evaluated the response to the α angle by investigating both a (2) narrow (conservative) range of $\alpha_{epi}$ and $\alpha_{endo}$ from −70 to −50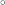 and 50 to 70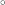, respectively, and a (3) broad (liberal) range of $\alpha_{epi}$ and $\alpha_{endo}$ from −100 to −20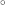 and 20 to 100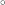, respectively. To evaluate the response of the β angle (4), we altered $\beta_{epi}$ and $\beta_{endo}$ uniformly between −10 to 10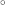 and −45 to −25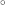, respectively, under conditions of constant α angles at the baseline values (Streeter et al., 1969; Lombaert et al., 2012). The four individual sets (as well as the baseline) of parameter distributions are summarized in Table 1.

### Eikonal simulations

All simulations of the activation sequence were computed with CARPentry using the eikonal approximation (Augustin et al., 2016; Neic et al., 2017; Vigmond et al., 2008), in which wavefront arrival times $t_{a}$ within the myocardium Ω are determined by a spatially heterogeneous orthotropic velocity field, represented by the tensor V(x), and the initial activation times $t_{0}$ at locations Γ. The governing equations for this model are expressed as follows:

(1) $$\left\{\begin{array}{ccc} & \sqrt{\nabla t_{a}^{T}V\nabla t_{a}}=1 & \text{in}\;\Omega ,\\ & t_{a}=t_{0} & \text{in}\;\Gamma , \end{array}\right.$$

where Ω represents the myocardial tissue, and $t_{a}$ is a positive scalar function that describes the time at which the activation wavefront arrives at the points x. The symmetric, positive‐definite tensor V(x) is a 3×3 matrix that encodes the squared velocities $\left(v_{l}\left(x\right),v_{t}\left(x\right),v_{n}\left(x\right)\right)$ corresponding to the fibre directions (l(x),t(x),n(x)), which are longitudinal, transverse and normal to the underlying sheet structure, respectively, that is,

(2) $$V=v_{l}^{2}ll^{T}+v_{t}^{2}tt^{T}+v_{s}^{2}nn^{T}.$$

As the velocity tensor V(x) is dependent on the fibre directions (l,t,s), the velocities $\left(v_{l},v_{t},v_{s}\right)$ must be proportionally related. For the entire myocardium, longitudinal conduction velocity was set to 100 cm/s (Good et al., 2018), and the transverse and normal to the underlying sheet structure velocities were defined as 2/3 and 1/3 of the longitudinal value, respectively (Taggart et al., 2000).

For each fibre orientation parameterization as specified above in Table 1, we stimulated each heart from nine locations that were anatomically equivalent across all the hearts: epicardial, mid‐myocardial and endocardial left ventricular free wall; epicardial and endocardial right ventricular free wall; epicardial and endocardial apex and epicardial anterior and posterior ventricular junction, as shown in Fig. 1. All locations were prescribed using universal ventricular coordinates (UVCs) (Bayer et al., 2018; Gillette et al., 2021) as defined in Table 2. The surfaces required to impose necessary boundary conditions on the simulations were automatically extracted using the open‐source software *meshtool* (Augustin et al., 2016).

**Table 2:** Nine stimulus sites described in UVCs. There were three sites in the same region of the left ventricular free wall: epicardial, mid‐myocardial and endocardial; two in the right free wall: epicardial and endocardial; two at the apex: epicardial and endocardial; and two at the ventricular junction: anterior and posterior. All locations were prescribed automatically using UVCs, z, ρ, ϕ and v

		z	ρ	ϕ	v
Left ventricle free wall	Epicardial stimulus	0.5	1.0	π	−1
	Mid‐myocardial stimulus	0.5	0.5	π	−1
	Endocardial stimulus	0.5	0.0	π	−1
Right ventricle free wall	Epicardial stimulus	0.5	1.0	0.0	1
	Endocardial stimulus	0.5	0.0	0.0	1
Apical	Epicardial stimulus	0.0	1.0	π	−1
	Endocardial stimulus	0.0	0.0	π	−1
Anterior ventricular junction	Epicardial stimulus	1.0	1.0	π/2	−1
Posterior ventricular junction	Epicardial stimulus	1.0	1.0	−π/2	−1

### Uncertainty quantification

We quantified the effects of uncertainty in the myocardial fibre orientation using the polynomial chaos expansion approach implemented in the open‐source software UncertainSCI (Narayan et al., 2023; Tate et al., 2023). Such parametric UQ generates a distribution of solutions based on a distribution of input parameters for a given model f. The model is parameterized by a j‐dimensional random variable p, such that $Y_{k}$ is the model output corresponding to the kth value of p, that is, $f\left(p_{k}\right)=Y_{k}$. Polynomial chaos expansion models f(p) by mapping p to outputs Y, and fits a polynomial emulator of degree d using N basis functions $\psi_{1},\cdots ,\psi_{N}$. This emulator allows for efficient analysis of the model's output distribution and provides key statistical moments like means, STDs and global sensitivities with respect to input parameter distributions. The advantages of the polynomial chaos expansion approach include its mathematical robustness and computational efficiency along with the availability of open‐source software tools (Narayan et al., 2023; Tate et al., 2023).

In this study, the polynomial chaos emulator was trained via randomized weighted least‐squares fitting using K training parameter samples, their associated weights and the model outputs. We used weighted approximate Fekete sampling (Bos et al., 2010; Guo et al., 2018), which provides the benefits of reduced sampling and computational overhead while stabilizing sample variability arising from randomization of the parameter samples. The primary input for weighted approximate Fekete sampling is the distribution over the parameters, represented as the probability density ν. Weighted approximate Fekete sampling begins with an initial set of $\tilde{G}$ randomly selected samples, denoted ${\tilde{p}}_{g}$, drawn from an importance sampling density $\nu_{N}$, which enhances stability and improves the least‐squares procedure during sample optimization (Narayan et al., 2023). This importance sampling density is optimal in the sense that it minimizes a certain stability factor arising from probabilistic analysis of the least squares procedure (Cohen & Migliorati, 2017). In the least‐squares setting, each sample is assigned a weight $w_{g}$, determined as the ratio of the probability density derivative in the original parameter space ν to that in the importance sampling density $\nu_{N}$. The number of samples $\tilde{G}$ and the weights $w_{g}$ are selected according to a prescribed failure probability tolerance 0<δ<1 and a relative accuracy parameter 0<ε<1 according to

(3) $${\tilde{p}}_{g}∼\nu_{N},\;w_{g}=\frac{d\nu ({\tilde{p}}_{g})}{d\nu_{N}\left({\tilde{p}}_{g}\right)},\;\tilde{G}\geq \frac{3\log(4N/\delta )}{\varepsilon^{2}}N.$$

Sampling in such a way using a least‐squares procedure probably attains near‐optimal accuracy guarantees (Cohen & Migliorati, 2017), but the samples generated in this way are random and so are subject to random fluctuations. An empirical strategy to mitigate these fluctuations that we leveraged is the weighted approximate Fekete sampling from (Guo et al., 2018). According to this strategy, the $\tilde{G}$ randomly chosen samples serve as the first step in solving a weighted D‐optimal design problem (determinant‐maximizing), which identifies the final K parameters. This is achieved by maximizing the volume through the following form:

(4) $$\max_{p_{k},p_{l}}\left(\det(D^{T}D)\right)\prod_{g=1}^{K}\frac{d\nu (p_{g})}{d\nu_{N}\left(p_{k}\right)},$$

where D is a K×N design matrix defined as $D_{k,n}=\psi_{p}\left(p_{k}\right)$. Once the optimal K parameters $p_{1},\cdots ,p_{K}$ are identified, their associated weights $w_{1},\cdots ,w_{K}$ are calculated using eqn (3). This sampling approach uses a least‐squares‐based method that offers provably near‐optimal accuracy guarantees (Cohen & Migliorati, 2017). While the samples are chosen randomly, similar to standard Monte Carlo methods, the specific procedure is constructed to reduce variability in accuracy and enhance stability in the results. A more complete description of weighted approximate Fekete sampling as applied to the polynomial chaos expansion is described by Narayan et al. (2023).

Previous applications of the UQ methodology in the context of cardiac propagation (Tanner et al., 2025) suggest that an essential first step is to establish the hyperparameters for UncertainSCI, mainly a suitable polynomial order and the number of samples from which to generate the emulator. To this end, in a previous study (Tanner et al., 2025), we evaluated polynomial orders ranging from 5 to 10, and their associated number of samples (higher order polynomials dictate more sample points). As the sample sets also include some degree of random selection, we identified six sample sets (three of the original sample size and three of a doubled sample size), which we implemented for each polynomial order. For our convergence analysis, we evaluated the statistical metrics from the UQ analysis across orders and numbers of samples to determine the minimal level of both that maintained rigorous mathematical guarantees. For the majority of cases presented here (three heart models and nine stimulus sites), a polynomial function emulator of degree 5 (31 samples) was sufficient to achieve consistent results. For three of the cases, we were required to increase either the order and/or sample size due, specifically (1) Geometry A for pacing at the epicardial posterior ventricular junction required polynomial order 6 (38 samples) when evaluating the influence of the α fibre direction, (2) Geometry C for endocardial apex stimulus required polynomial order 6 (38 samples) when evaluating the α fibre direction and (3) Geometry A for mid‐myocardial left ventricular free wall stimulus required polynomial order 6 with double the amount of samples (76 samples) when evaluating the broader range of the α fibre direction. The hyperparameters were not always ideal, as evidenced by maximum residuals of 10.93, 42.58 and 27.00 for Cases 1, 2 and 3, respectively, compared to values below 1 for other stimuli and geometries. This variability is an inevitable aspect of UQ emulators that requires some degree of trial and error.

### Statistical and activation sequence metrics

The useful products of a UQ analysis are statistical moments that capture the effects of parameter uncertainty on model output. To illustrate this process, we calculated the mean, STD, global sensitivities and residuals for epicardial activation times. Global sensitivities quantify the relative contribution of each uncertain input parameter to the overall variability observed in the model outputs. Unlike local sensitivity measures that examine changes around a single point, global sensitivities consider the full range of input variability, providing a comprehensive view of which parameters most influence model behaviour.

**Figure 2:**
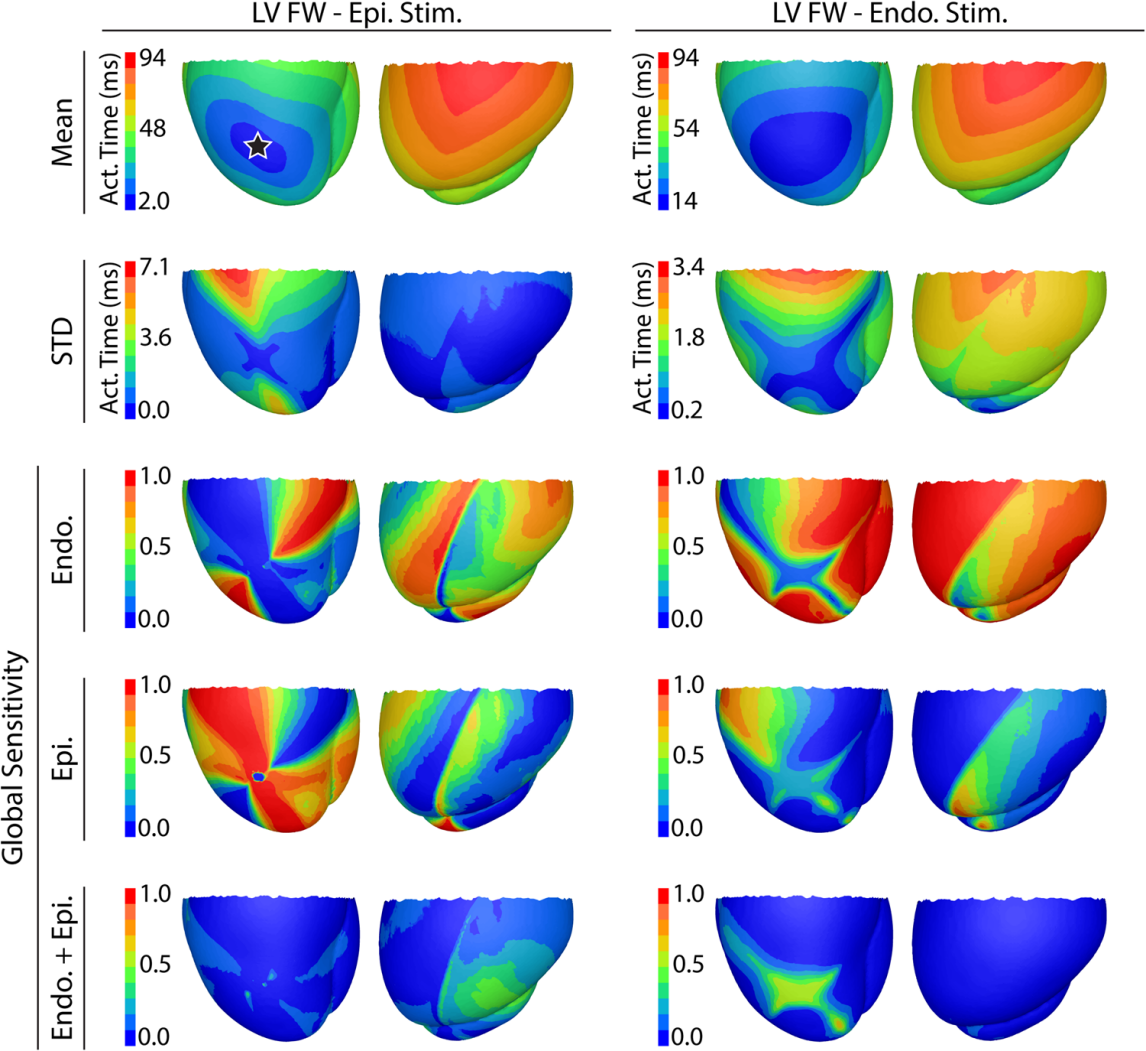
Uncertainty quantification of epicardial activation times following left ventricular free wall stimuli on Geometry A The left panels in each row show the response to epicardial pacing from the left ventricular free wall, while the right panel shows the response to endocardial pacing from the same region of the left ventricle. Row 1 shows the mean activation sequence; Row 2 shows the STD; Rows 3–5 show the global sensitivity contributions of variations in the endocardial (Row 3), epicardial (Row 4) and combined endocardial and epicardial (Row 5) fibre orientations, respectively. The perspectives in all pairs of figures are left and right lateral views, respectively, along the long axis of the heart. The star (located on the mean) represents the location of the epicardial point stimulus used in the simulations. Geometries B and C showed similar results.

In addition, we analysed the statistics of three metrics quantifying the activation sequence: (1) the volumetric total activation time, (2) the location of the earliest epicardial activation (breakthrough) for endocardial and mid‐myocardial stimuli and (3) the location of the latest activation on the epicardial surface. Furthermore, we evaluated two additional metrics quantifying the activation sequence for all the left ventricular free wall stimuli: (1) the area and (2) the orientation of the epicardial early activation isochrone (10% of the epicardial total activation time). The epicardial early activation isochrone is visually represented by the dark blue region in the colour map, as shown in Fig. 2. We calculated the orientation from the projection of this isochrone onto a plane fit to the nearby segment of the epicardial surface. We then implemented a set of conic equations to fit an ellipse to these projected points and identified the direction of its major axis relative to the long axis of the heart, following an approach proposed by Taccardi et al. (2008). The area and orientation of the epicardial early activation isochrone have been shown in previous studies by Taccardi et al. to relate to fibre orientation and pacing depth, which is why we included these metrics in our analysis. Finally, for the left ventricular free wall stimuli, we also visualized the activation times and associated UQ parameters, mean, STD and global sensitivities, on the endocardial surface.

## Results

### Alpha angle

#### Activation patterns on epicardial and endocardial surfaces

**Left ventricular free wall stimuli**: Figure 2 contains epicardial renderings of the mean, STD and global sensitivity following epicardial and endocardial left ventricular free wall stimuli, given variations in the epicardial and endocardial alpha fibre angle. For both stimuli, the activation sequences for both baseline (not shown) and mean fibre orientations had similar patterns and ranges. For the epicardial stimulus, the STD values were very small (0.0 ms) at the stimulus site and termination regions and grew higher (6.8–7.1 ms across all three geometries) near the apex and left lateral basal regions of the ventricles. For the endocardial stimulus, the STD patterns were more complex than for the epicardial stimulus, although lower in amplitude (3.1–3.7 ms across all three geometries). They were low near the breakthrough location (0.2–0.4 ms) and grew towards the left and right lateral bases. The global sensitivities for the epicardial left ventricular free wall stimuli were different for epicardial and endocardial fibre variations, almost orthogonal in their patterns. The interaction terms (Endo. + Epi.) of sensitivity were generally very low, suggesting minimal statistical contributions to the STD values. The global sensitivities for the endocardial stimulus showed similarly orthogonal or complementary patterns with generally low interactions other than perhaps near the breakthrough location. In all cases, it is possible to view the STD as a reflection of the combined effects of the global sensitivities of the two sources of uncertainty.

**Figure 3:**
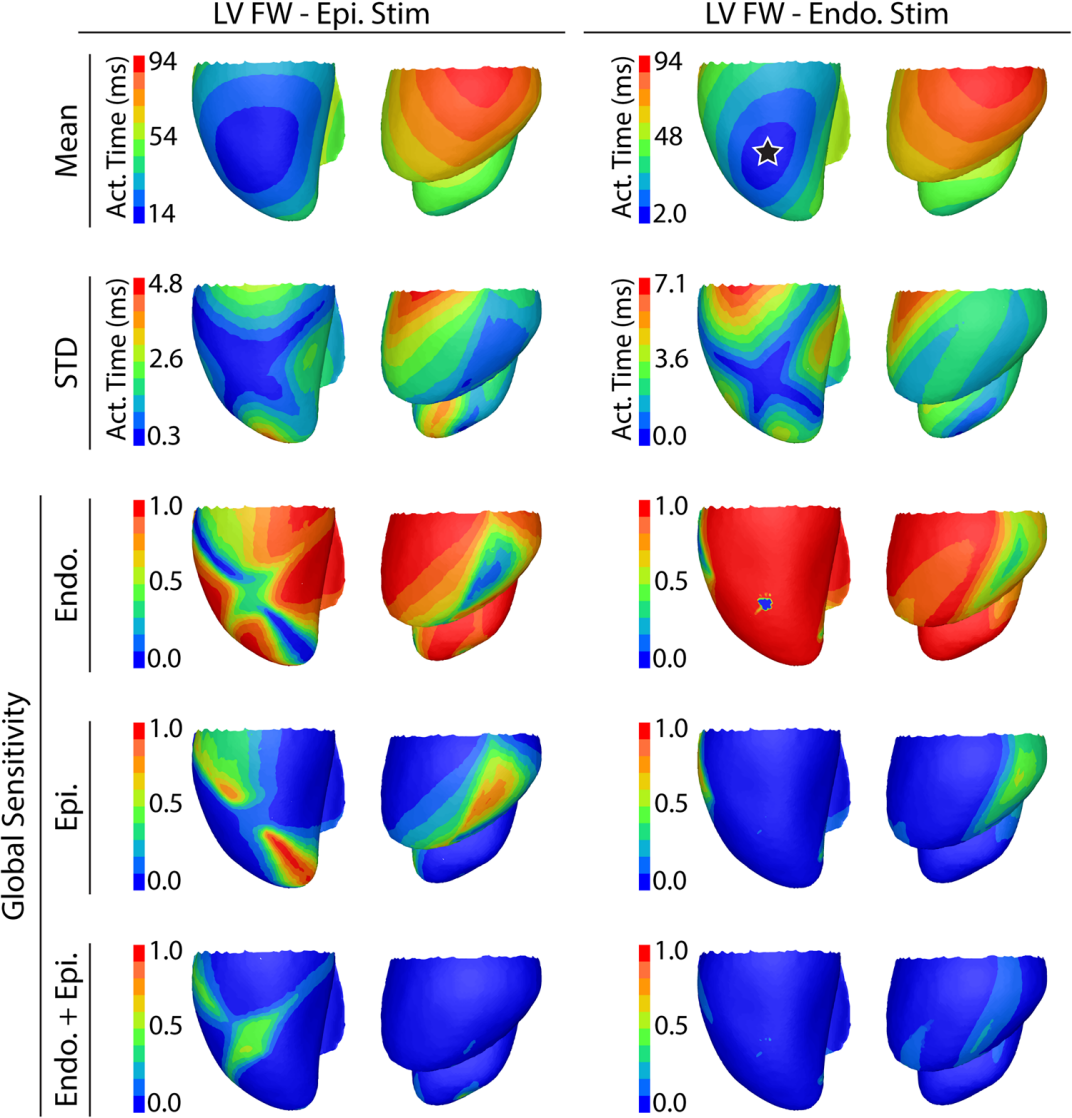
Uncertainty quantification of endocardial activation times following left ventricular free wall stimuli on Geometry A The left panels in each row show the response to epicardial pacing from the left ventricular free wall, while the right panel shows the response to endocardial pacing from the same region of the left ventricle. The rows contain the same quantities as in the previous figure, displayed from similar perspectives but on the endocardial surfaces. The star (located on the mean) represents the location of the endocardial point stimulus used in the simulations. Geometries B and C showed similar results.

Figure 3 shows equivalent renderings of the mean, STD and global sensitivities as in Fig. 2 of the epicardial and endocardial left ventricular free wall stimuli, but now displayed on the endocardial surfaces. As before, the baseline (not shown) and mean activation sequences had similar activation time patterns and ranges. The STD patterns were similar for both pacing sites, but larger for the endocardial stimuli, ranging between 6.7 and 7.1 ms compared to 3.9 and 4.8 ms across all three geometries near flanking regions of the stimulus site, down to approximately 0.0 ms across all three geometries, in a cross pattern around the stimulus site. The global sensitivities once again showed a complementary structure between the epicardial and endocardial fibre effects, with the endocardial fibres dominating except in regions with low STD, where they both contributed.

**Figure 4:**
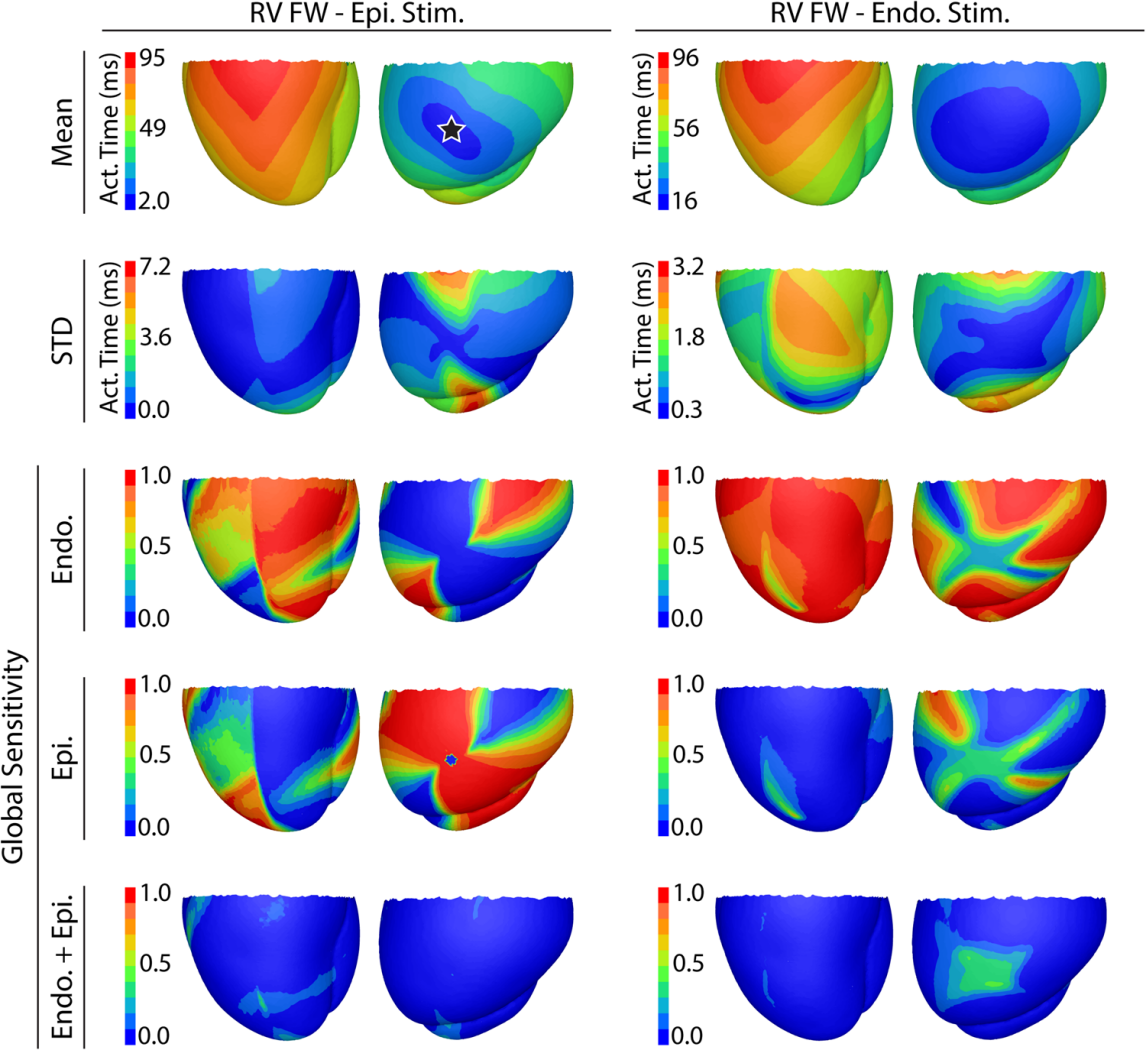
Uncertainty quantification of epicardial activation times following right ventricular free wall stimuli on Geometry A The left panels in each row show the response to epicardial pacing from the right ventricular free wall, while the right panel shows the response to endocardial pacing from the same region of the right ventricle. The rows are organized as in the previous figures. The star (located on the mean) represents the location of the epicardial point stimulus used in the simulations. Geometries B and C showed similar results.

**Right ventricular free wall stimuli**: Figure 4 shows equivalent response to variation in the alpha fibre angle for epicardial and endocardial right ventricular free wall stimuli, visualized on the epicardial surfaces. The stimuli resulted in similar mean patterns of activation (top row), relative to the stimulation site, as the epicardial left ventricular free wall stimuli in Figure 2. As before, the baseline (not shown) and mean activation sequences had similar activation time patterns and ranges. The STD values were higher for the epicardial stimulus, ranging between a maxima of 5.1 and 7.2 ms across all three geometries located near flanking regions of the stimulus site and approximately 0.0 ms directly over the stimulus site and across the left ventricle. For the endocardial stimulus, the maximum STD peaked around 3.0–3.3 ms across all three geometries at the right ventricle apex and base and on the left ventricle free wall. The STD was low, approximately 0.3 ms across all three geometries, around the right ventricle breakthrough site. The global sensitivities showed patterns that were similarly complementary as in previous figures, but with markedly different spatial distributions.

**Figure 5:**
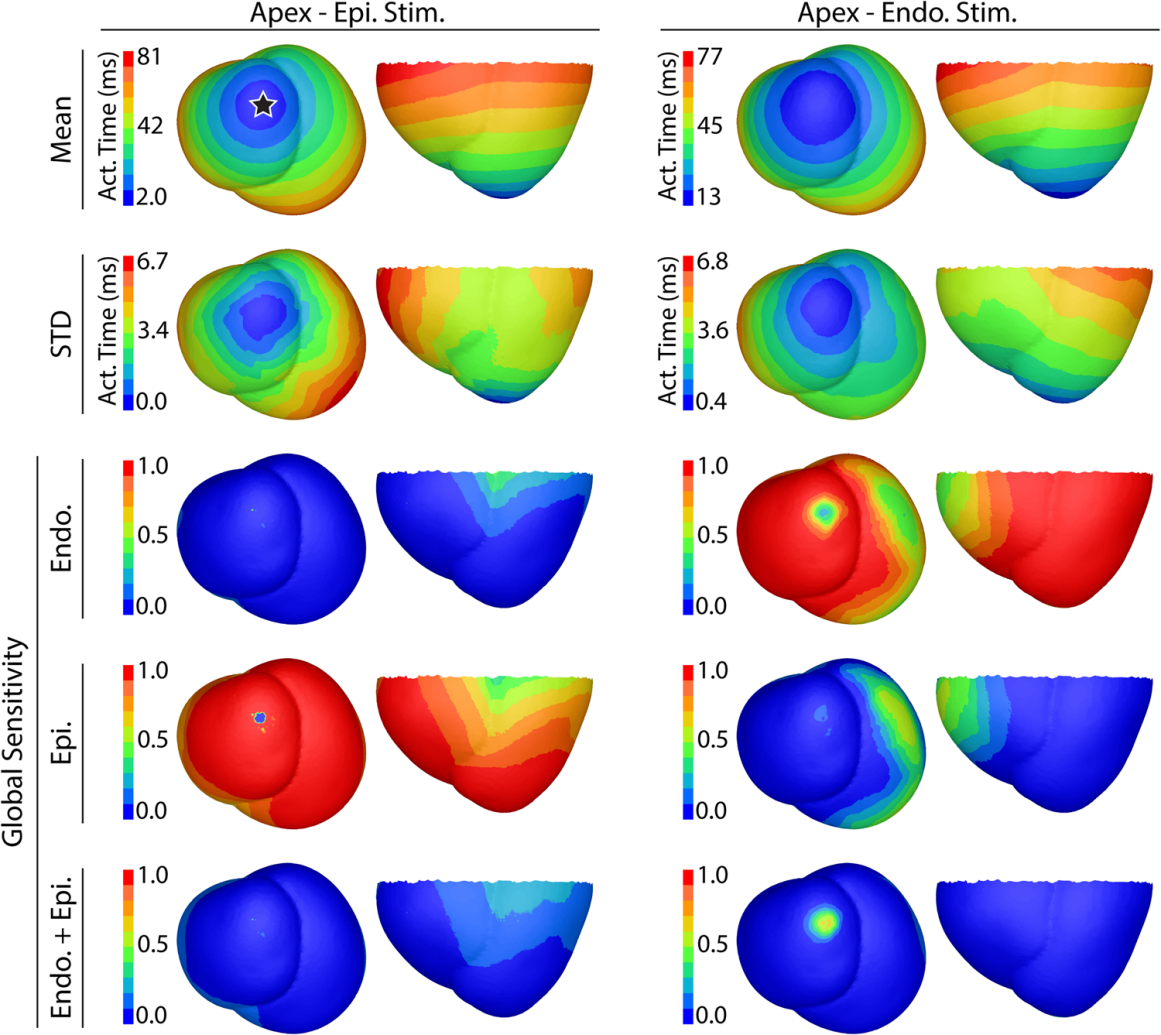
Uncertainty quantification of epicardial activation times following apical stimuli on Geometry A The left panels in each row show the response to epicardial pacing from the apex, while the right panel shows the response to endocardial pacing from the same region of the apex. The rows are organized as in the previous figures. The perspectives in all pairs of figures are apical and anterior views, respectively. The star (located on the mean) represents the location of the epicardial point stimulus used in the simulations. Geometries B and C showed similar results.

**Apical stimuli**: Figure 5 shows the equivalent responses to variability in the alpha fibre angle for epicardial and endocardial apex stimuli on the epicardial surface. For both stimuli, the baseline (not shown) and mean activation sequences had similar activation time patterns and ranges. The STD values were very similar for both stimuli, but the patterns were somewhat orthogonal. For the epicardial stimulus, the largest values of STD were located on the basal free wall of the right ventricle, while those for endocardial stimuli were in a symmetrical region of the left ventricle. The global sensitivities for endocardial and epicardial variability were generally complementary, as seen in other cases. However, for the epicardial stimulus, both the endocardial and epicardial fibre angles contributed to the variability on the basal anterior surface near the ventricular junction. For endocardial stimuli, the region of shared contributions to variability was on the right ventricular free wall, again near the base. Striking was the very low global sensitivity from both fibre angles directly over the stimulus location. At these locations, there was strong interaction between the endocardial and epicardial fibre angles, as indicated in the focal elevated points seen in the last row of the figure.

**Figure 6:**
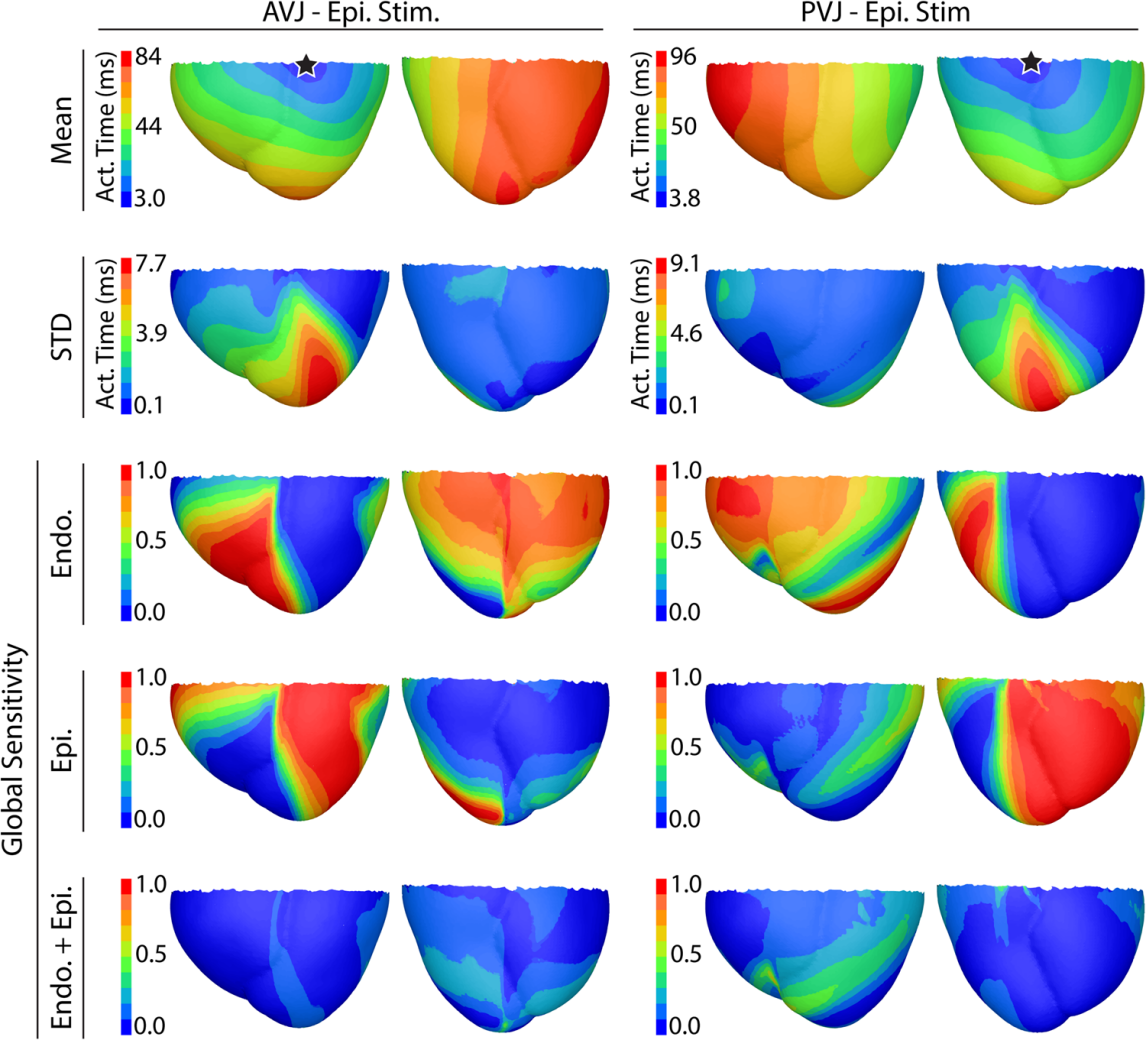
Uncertainty quantification of epicardial activation times following ventricular junction stimuli on Geometry A The left panels in each row show the response to epicardial pacing from the anterior ventricular junction, while the right panel shows the response to epicardial pacing from the posterior junction. The rows are organized as in the previous figures. The perspectives in all pairs of figures are the anterior and posterior views, respectively. The star (located on the mean) represents the location of the epicardial point stimulus used in the simulations. Geometries B and C showed similar results.

**Ventricular junction stimuli**: Figure 6 shows equivalent responses to variation in alpha fibre angles for the anterior and posterior ventricular junction stimuli on the epicardial surfaces. As for all other cases, the baseline (not shown) and mean activation sequences had similar activation time patterns and ranges. The STDs for both stimuli were similar in value (7.7–9.5 vs. 8.5–9.1 ms) and symmetric in overall pattern. The regions of large STD were aligned with the pacing locations, that is, anteriorly for the anterior ventricular junction stimulus and posteriorly for the posterior ventricular junction stimulus. The global sensitivities once again showed very complementary patterns for endocardial and epicardial fibre orientations and only small regions of interactions. In both cases, the epicardial fibre variability dominated the contribution, leading to the maximum STD with moderate effects from the endocardial fibres.

#### Evaluation of activation sequence metrics

**Left ventricular free wall stimuli**: The total volumetric activation times were very comparable, with means ranging between 96–104, 94–102 and 97–105 ms, and STDs ranging between 0.9–1.7, 1.7–2.0 and 1.8–2.7 ms, for the epicardial, mid‐myocardial and endocardial stimuli, respectively. There was minimal displacement of the locations of the breakthrough sites for the mid‐myocardial and endocardial left ventricular free wall stimuli, with a maximum displacement of 0.66 mm across all three geometries. Similarly, the locations of the latest sites of activation for the epicardial, mid‐myocardial and endocardial stimuli showed minimal displacement with fibre variation, shifting only by 1.41, 1.84 and 2.67 mm, respectively.

**Right ventricular free wall stimuli**: The total volumetric activation times were very comparable, with means ranging between 96–99 and 98–102 ms, and STDs ranging between 0.7–1.8 and 0.9–2.8 ms, for the epicardial and endocardial stimuli, respectively. The locations of breakthrough sites were also very stable, with a maximum displacement of only 0.49 mm across the three geometries. Similarly, the location of the latest site of activation for the epicardial and endocardial stimulus had minimal displacement with maxima of 1.02 and 1.39 mm, respectively.

**Apical stimuli**: The mean total volumetric activation times were similar for both pacing sites, ranging between 86–99 and 85–97 ms, while the STD values differed clearly, ranging between 4.5–4.9 and 1.5–3.0 ms, for the epicardial and endocardial stimuli, respectively. There was minimal displacement of the location of the breakthrough site for the endocardial apex stimulus, with a maximum displacement of 1.12 mm across the three geometries. However, the location of the latest site of activation for the epicardial and endocardial stimuli had more substantial displacements with maxima of 9.33 and 6.95 mm, respectively.

**Ventricular junction stimuli**: The total volumetric activation time was slightly lower for the anterior stimulus (85–97 vs. 97–100 ms for posterior), while the STD values were practically the same (1.5–3.0 and 1.4–2.5 ms for the anterior and posterior stimuli, respectively). For the location of the latest site of activation, there was substantial displacement for both anterior and posterior stimuli, much larger for the anterior stimulus (18.20 vs. 6.24 mm).

#### Epicardial early activation isochrone metrics

As expected, the mean area of the epicardial early activation isochrone (10%) increased for all three geometries with distance from the stimulus site, ranging between 185 and 206, 377 and 436 and 675 and 758 mm^2^, for the epicardial, mid‐myocardial and endocardial left ventricular free wall stimuli, respectively. A similar response was documented by the STD for the area of the epicardial early activation isochrone, which ranged between 7.7 and 9.8, 27.0 and 34.2 and 53.8 and 65.3 mm^2^, for the epicardial, mid‐myocardial and endocardial left ventricular free wall stimuli, respectively.

Table 3 contains the statistics for the orientation of the epicardial early activation isochrone given variable fibre orientation ranges, that is, for the epicardial fibre, the narrow range was between −70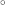 and −50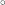, the physiological range was between −85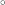 and −35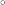 and the broad range was between −100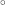 and −20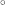. The mean epicardial early activation isochrone orientation had minimal variability across the three fibre ranges. On the contrary, the STD increased with the fibre range for each pacing site, but did not respond uniformly with distance from the stimulus site as the area did. Not surprisingly, the global sensitivity was tied to the stimulus site, that is, variability for the epicardial stimulus corresponded primarily to the epicardial fibre parameter and variability for the endocardial stimulus to the endocardial fibres. As one would expect, the global sensitivity for the mid‐myocardial stimulus corresponded to a combination of both the epicardial and endocardial fibre parameters.

**Table 3:** Statistics for the epicardial early activation isochrone orientation given variable fibre orientation ranges. The first column corresponds to each stimulus location: epicardial, mid‐myocardial and endocardial. For each stimulus, the table contains the mean and STD (Row 1), the global sensitivity for the endocardial fibre parameters (Row 2) and the global sensitivity for the epicardial fibre parameters (Row 3). The last three columns correspond to the fibre range, specifically the narrow range, physiological range and broad range

		Narrow range	Physiological range	Broad range
Epi. Stim.	Mean ± STD	−50.1 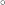 ± 5.2 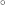	−51.2 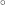 ± 14.2 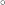	−48.8 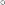 ± 26.3 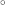
	Glob. Sens. Endo.	0.04	0.01	0.03
	Glob. Sens. Epi.	0.90	0.98	0.84
Mid. Stim.	Mean ± STD	−9.9 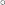 ± 4.5 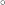	−9.8 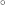 ± 10.6 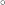	−7.5 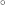 ± 11.0 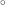
	Glob. Sens. Endo.	0.57	0.32	0.59
	Glob. Sens. Epi.	0.41	0.64	0.38
Endo. Stim.	Mean ± STD	23.8 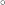 ± 6.5 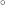	27.0 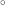 ± 12.8 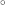	28.8 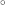 ± 19.6 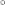
	Glob. Sens. Endo.	0.91	0.95	0.96
	Glob. Sens. Epi.	0.03	0.02	0.01

### Beta angle

For all stimuli, the baseline and mean epicardial activation sequences had similar activation time patterns and ranges regardless of the beta fibre angle. There was also only minimal variation, with STD across all stimuli between 0.0 and 1.7 ms (see Table 4). As variations in the beta angle were not influential, no visual representation is provided.

**Table 4:** Range of activation time variability (standard deviation in milliseconds) across nine stimulus sites due to changes in the beta fibre angle. Values represent the minimum and maximum standard deviation observed for each stimulus configuration

		Standard deviation (ms)	
		Minimum	Maximum
Left ventricle free wall	Epicardial stimulus	0.0	1.4
	Mid‐Myocardial stimulus	0.5	1.4
	Endocardial stimulus	0.1	1.0
Right ventricle free wall	Epicardial stimulus	0.0	0.8
	Endocardial stimulus	0.0	1.0
Apical	Epicardial stimulus	0.0	1.2
	Endocardial stimulus	0.2	0.9
Anterior ventricular junction	Epicardial stimulus	0.0	0.9
Posterior ventricular junction	Epicardial stimulus	0.0	1.7

## Discussion

Myocardial fibres govern the spread of excitation in the heart; however, the current understanding of the extent and degree to which propagation varies in response to fluctuations in fibre orientation is incomplete (Taccardi et al., 2008; Lombaert et al., 2012). Furthermore, predictive models and simulations of cardiac function require accurate representations of anatomy, often to the scale of local myocardial fibre structure (Bayer et al., 2018; Gillette et al., 2022). This study aimed to evaluate the role of variations in fibre orientation on activation sequences using robust uncertainty quantification algorithms. We implemented biventricular eikonal simulations, rule‐based algorithms to generate fibre orientations and polynomial chaos expansion techniques to generate detailed quantitative statistics (Vigmond et al., 2008; Bayer et al., 2018; Gillette et al., 2021; Narayan et al., 2023; Tate et al., 2023). As outlined in the methods, applying polynomial chaos expansion requires a thorough evaluation of the associated hyperparameters, which were carefully investigated to ensure the reliability of the output statistics (Tanner et al., 2025).

For all variations in fibre angles, the activation maps for all stimuli showed highly similar baseline and mean activation sequences, suggesting that baseline outputs for fibre orientation and activation on the epicardial and endocardial surfaces are good approximations for first‐order (mean) statistics under our uncertainty model.

Another recurring finding was the lack of substantial parameter interactions indicated by the combined global sensitivity. For all stimuli, the endocardial and epicardial global sensitivities were complementary, for example, in regions where the endocardial sensitivity was approximately 1.0, the epicardial sensitivity was approximately 0.0 and vice versa. This result was unexpected as changing the epicardial or endocardial fibre parameters changes the fibre orientation throughout the entire wall via a relatively linear function (Bayer et al., 2012; Streeter et al., 1969). Thus, mathematically, the rule‐based fibre algorithm creates a dependence between the epicardial and endocardial fibres. However, this relationship did not reflect a corresponding interaction in the activation patterns we observed.

For the free wall stimulus sites, three trends in activation patterns emerged (Figs 2, 3, 4): (1) The amplitudes of the STD were highest on the surface that was stimulated, that is, both right‐ and left‐ventricular epicardial stimulation resulted in consistently higher STD values on the epicardial surface, while endocardial stimulation produced the reverse, higher STD values on the endocardial surface. In all cases, these differences were substantial, for example, in some cases, twice as high on the stimulation surface as on the remote surface. These findings suggest that deviations in the early phase of activation are most pronounced near the stimulus site and diminish as the activation propagates to the remote surface. This is a somewhat counterintuitive finding, as one would expect deviations that are set up early in the activation sequence to spread and become even larger on the remote surface, as one sees in the activation maps themselves. (2) The shapes of the STD patterns had less variation on the remote surfaces than on the stimulus surfaces. This finding does match the intuition of deviation spreading with time and distance from the stimulus, even though the maximum STD values were consistently smaller. (3) The peak values of STD were highest in areas approximately orthogonal to the local fibre orientation near both the stimulus and breakthrough sites. A possible explanation for this finding could be attributed to the collision of wavefront profiles, as previously described by Franzone et al. (1998) and Taccardi et al. (2008). Briefly, as the activation front spreads away from the stimulus site through the rotating fibres, some portions that dive into the ventricular wall rotate and then overtake the superficial wavefront in regions where this excitation spreads slowly across the fibres. As a consequence, some intramural portions of the wavefront can reach the stimulus surface in areas orthogonal to the longitudinal direction of the primary superficial fibre orientation, resulting in the intersection of the wavefront at the surface.

For the apical stimulus sites, two intriguing trends emerged (Fig. 5): (1) Similar to the free wall stimuli, the parameter global sensitivity on the stimulus surface strongly dominated the STD, that is, Rows 3 and 4 in the figure. In other words, the variability in fibre orientation on the stimulus surface dominates the variability in the activation sequence, in this case, even when the amplitudes of STD were the same for both stimulus surfaces. (2) While the mean activation patterns for both epicardial and endocardial stimulus were very similar, the patterns of STD showed complementary locations of maximal values, that is, epicardial pacing produced the largest STD on the **right** basal area while endocardial pacing resulted in maximal STD on the **left** base. This finding suggests a highly labile dependence on fibre structure for apically stimulated beats, with possible implications for cardiac re‐synchronization therapy (Kutyifa et al., 2013).

Results for the ventricular junction stimulus sites (Fig. 6) also reflected the complex and poorly documented fibre structure at this junctional region (Streeter et al., 1969). Both anterior and posterior stimuli produced symmetrical early activation that shared the same lateral path from the left to the right sides of the heart. The STD patterns were similarly symmetric, with low values near each stimulus site, but with a rapid transition to large values near the apex. Similar to previous findings, the larger STD was localized to the same aspect as the stimulus, that is, anterior stimulation resulted in high STD on the anterior side of the apex. For both stimulus locations, the largest STD values were on the left side of the junction in the apical region. Given the uncertain nature of fibre structure at these junctions, it is necessary to interpret the results with great care, as they could be a product of the rule‐based assignment of fibres rather than a reflection of robust physiology (Bayer et al., 2012).

For the three metrics quantifying the activation sequence, for all stimuli we observed the following three trends: (1) the total volumetric activation time varied minimally with fibre orientation with a maximum STD of 4.9 ms; (2) similarly, the location of the epicardial breakthrough sites (for mid‐myocardial and endocardial stimuli) showed minimal dependence on fibre orientation with a maximum STD of only 1.12 mm and (3) by contrast, the location of the latest site of activation showed high variability with fibre orientation with a maximum STD of 18.20 mm. These findings suggest that fibre orientation plays a minimal role in the duration of ectopic activation or the location of discrete activation features relatively early in the sequence but creates substantial variability in the pattern of activation late in the sequence.

For the two metrics quantifying the early phase of the activation sequence following left ventricular free wall stimuli at variable depths (Table 3), we observed the following three trends: (1) the STD increased as the stimulus depth increased for the epicardial early activation isochrone area; (2) the orientation of the epicardial early activation isochrone showed variability that grew rapidly with the range of fibre directions (fivefold increase) and (3) the orientation of the epicardial early activation isochrone was sensitive to variability of the epicardial fibre direction for epicardial stimuli and sensitive to the endocardial fibre direction for endocardial stimuli. Our results match previous findings (Taccardi et al., 2008; Muzikant et al., 2002; Franzone et al., 1998; Johnston et al., 2018), which suggested that as pacing depth increases, the orientation of the epicardial early activation isochrone rotates counterclockwise in the left ventricle free wall. Our results expand on previous reports in the observation that mid‐myocardial pacing generates activation patterns that are the least sensitive to fibre orientation. We suggest that the strong response of activation to the local fibre direction for epicardial or endocardial pacing arises because of the close proximity of early activation and regions of variable fibre orientation. Mid‐myocardial stimulus, by contrast, was equally affected by both endocardial and epicardial fibre variation in a way that, to some extent, cancelled the two effects out. The small increase in STD between stimulation from the epicardial *versus* endocardial surfaces, at least for the physiological and broad range of fibre orientations, likely arose because the epicardially paced wavefront travels through such a small number of fibres before reaching the epicardium. Therefore, the orientation of the early activation isochrone closely follows that of the superficial epicardial fibres. However, endocardial pacing sites produce activations that must travel through the entire wall before reaching the epicardial surface, thus experiencing what appears to be a smoothing or averaging effect.

We have shown how uncertainty quantification provides a complex and rich output when applied to cardiac simulations. A thorough uncertainty quantification analysis has distinct advantages over the approaches used in most previously reported studies that have sought to establish the effects of fibre orientation on cardiac activation (Taccardi et al., 2008; Muzikant et al., 2002; Franzone et al., 1998). Most notably, rather than investigating only a small subset of model parameter values, in an uncertainty analysis that uses rule‐based fibres and a sophisticated UQ approach, one is able to explore the impact of a continuous range of fibre orientations on activation times. In addition, this approach provides more detailed spatial information than simplified models used in most other investigations of this type (Johnston et al., 2018; Quaglino et al., 2018). Most generally, while mean values provide some insight into parameter sensitivity, this type of UQ analysis can produce additional statistical moments, particularly STD and global parameter sensitivities.

Furthermore, fibre orientation represents only one of many parameters influencing cardiac electrophysiology simulations. A growing body of work has explored uncertainty quantification in cardiac modelling, with some studies using relatively simple techniques (Pathmanathan et al., 2019; Dhamala et al., 2018) and others adopting more advanced and rigorous UQ methodologies (Zaman et al., 2021; Hu et al., 2018). Although many of these investigations do not focus specifically on fibre orientation, they underscore the broader scientific interest in understanding how parameter uncertainty affects model predictions. This growing emphasis highlights a clear need for comprehensive UQ analyses to evaluate the variability and potential hidden impacts of uncertain model parameters across all aspects of cardiac simulations.

### Limitations

Our models used rule‐based fibre orientations, which can be seen as both a limitation and a strength. Rule‐based approaches have already shown their utility, but they are still an approximation. However, they are a parameterized description of fibre orientation, which allowed us to impose a carefully controlled range of fibre orientations. Subject‐specific heart models using image‐based modalities could be more accurate for a single heart, but adjusting fibre directions in a systematic way requires assumptions for which the data are inadequate (Bayer et al., 2018). Moreover, rule‐based methods offer a level of consistency across models that is essential for comparative studies, especially when exploring sensitivity to fibre orientation. They also enable efficient generation of large model cohorts without the logistical and technical barriers associated with acquiring high‐resolution imaging for each case. In addition, noise is present in rule‐based fibre orientations, particularly in ill‐defined regions such as the ventricular junction, where the commonly used assumption of linear transmural variation in fibre angles may fail to capture the true structural complexity and heterogeneity of the myocardium, challenging precise parameterization. This could influence the physiological realism of our simulations, though such effects are difficult to quantify without more detailed and widely available imaging data.

An additional possible source of error in our simulations is the homogeneous conductivity velocity tensor we assigned, which may mask more complex activation patterns. Furthermore, our computational models lacked an explicit His–Purkinje system, which could alter the early phases of endocardial stimulation. However, such models are also based on limited data and remain a source of active research (Gillette et al., 2021). The inclusion of a fast‐conducting endocardial layer or a modelled His–Purkinje system could lead to earlier and more synchronized epicardial breakthrough, particularly affecting the timing and pattern of early activation. This may, in turn, reduce the observed variability attributed to fibre orientation in our results. However, incorporating these features would require detailed anatomical and physiological data that are not consistently available across all specimens, and doing so would introduce additional uncertainty into the model.

A further limitation is that the simulations were based on the eikonal simplification rather than a full bidomain formulation, but previous studies (Neic et al., 2017) indicate that the eikonal and full bidomain produce comparable results in this setting. The eikonal model provides a computationally efficient approximation of activation timing by solving for the fastest arrival of depolarization fronts, but it does not account for the full biophysical detail of transmembrane currents or extracellular potential distributions. While this simplification is appropriate for modelling the spread of activation in healthy tissue during ectopic stimuli, it may be less suitable for capturing the effects of electrical interactions between neighbouring cells or conduction abnormalities that could arise in more complex or pathological settings. Nevertheless, for the purpose of quantifying variability in activation due to fibre orientation under controlled conditions, the eikonal approach offers a practical and validated framework.

### Conclusion

Our findings suggest that uncertainties in fibre orientation can affect features of the resulting activation sequences, but in complex ways that depend on the pacing site and the metric or feature of interest in the study. We observed the most variability during the middle and late phases of the spread of activation, which would be especially meaningful in the modelling of re‐entrant arrhythmias, possibly resulting in differences in the site or even the existence of re‐entry. Furthermore, such modelling applications related to re‐entry or other complex electrical pathways may require patient‐specific fibre orientation and the inclusion of disease‐related substrate changes to carry out accurate simulations. Future studies will explore a broader range of arrhythmias to improve our understanding of fibre orientation variability in predicting and localizing abnormal electrical activity in the heart.

## Additional information

## Competing interests

The authors have no relevant financial or non‐financial interests to disclose.

## Author contributions

L.C.R.T., K.G., J.A.B., W.W.G., B.Z., A.N., G.P. and R.S.M. have contributed to the conception or design of the work. L.C.R.T, A.B., K.G., J.A.B., A.N. and R.S.M. have contributed to the acquisition, analysis or interpretation of data for the work. All authors have contributed to the work, either by drafting the manuscript or critically revising it for important intellectual content. They have all approved the final version of the manuscript. Each author agrees to be accountable for all aspects of the work, ensuring that any questions related to the accuracy or integrity of the work are thoroughly investigated and resolved. Furthermore, all individuals who qualify for authorship are included as authors, and all listed authors meet the established criteria for authorship.

## Funding

The research leading to these results received funding from the National Institutes of Health under grant agreements P41 GM103545, R24 GM136986, T32 HL007576 and U24EB029012; the National Science Foundation Graduate Research Fellowship Program and the Nora Eccles Harrison Foundation for Cardiovascular Research.

## Supporting information


Peer Review History

## Data Availability

The simulated data that support the findings of this study are not publicly available but are available from the corresponding author upon reasonable request.
