# Supplementary material for: Uncertainty quantification via polynomial chaos expansion of myocardial fibre orientation and cardiac activation patterns
Source: J Physiol. 2025 Aug 22;604(13):5440–57. doi: 10.1113/JP287746 (PMC13327771; doi:10.1113/JP287746)
Supplement: Supplementary file 1 — Peer Review History [file TJP-604-5440-s001.pdf]

The Journal of Physiology

Uncertainty Quantification via Polynomial Chaos Expansion of Myocardial Fiber Orientation and Cardiac Activation Patterns

Lindsay C.R. Tanner, Anna Busatto, Jake A Bergquist, Wilson W Good, Brian Zenger, Gernot Plank, Akil Narayan, Karli Gillette, and Rob S. MacLeod
DOI: 10.1113/JP287746

Corresponding author(s): Lindsay Tanner (lindsay.c.r.tanner@utah.edu)

The following individual(s) involved in review of this submission have agreed to reveal their identity: Caroline H Roney (Referee #2)

Review Timeline:

Submission Date:	18-Jan-2025
Editorial Decision:	19-May-2025
Revision Received:	07-Jun-2025
Accepted:	11-Jul-2025

Senior Editor: Bjorn Knollmann

Reviewing Editor: Viviane Timmermann

Transaction Report:

(Note: With the exception of the correction of typographical or spelling errors that could be a source of ambiguity, letters and reports are not edited. Depending on transfer agreements, referee reports obtained elsewhere may or may not be included in this compilation. Referee reports are anonymous unless the Referee chooses to sign their reports.)

Dear Dr Tanner,

Re: JP-RP-2025-287746 "Uncertainty Quantification of Fiber Orientation and Cardiac Activation Patterns" by Lindsay C.R. Tanner, Anna Busatto, Jake A Bergquist, Wilson W Good, Brian Zenger, Gernot Plank, Akil Narayan, Karli Gillette, and Rob S. MacLeod

Thank you for submitting your manuscript to The Journal of Physiology. It has been assessed by a Reviewing Editor and by 1 expert referee and we are pleased to tell you that it is potentially acceptable for publication following satisfactory major revision.

Please advise your co-authors of this decision as soon as possible.

The referee reports are copied at the end of this email.

Please address all the points raised and incorporate all requested revisions or explain in your Response to Referees why a change has not been made. We hope you will find the comments helpful and that you will be able to return your revised manuscript within 2 months. If you require longer than this, please contact journal staff: jp@physoc.org. Please note that this letter does not constitute a guarantee for acceptance of your revised manuscript.

Your revised manuscript should be submitted online using the link in your Author Tasks: Link Not Available. This link is accessible via your account as Corresponding Author; it is not available to your co-authors. If this presents a problem, please contact journal staff (jp@physoc.org). Image files from the previous version are retained on the system. Please ensure you replace or remove any files that are being revised.

If you do not wish to submit a revised version of your manuscript, you must inform our journal staff (jp@physoc.org) or reply to this email to request withdrawal. Please note that a manuscript must be formally withdrawn from the peer review process at one journal before it may be submitted to another journal.

TRANSPARENT PEER REVIEW POLICY: To improve the transparency of its peer review process, The Journal of Physiology publishes online as supporting information the peer review history of all articles accepted for publication. Readers will have access to decision letters, including Editors' comments and referee reports, for each version of the manuscript, as well as any author responses to peer review comments. Referees can decide whether or not they wish to be named on the peer review history document.

ABSTRACT FIGURES: Authors are expected to use The Journal's premium BioRender account to create/redraw their Abstract Figures. Information on how to access this account is here: https://physoc.onlinelibrary.wiley.com/journal/14697793/biorender-access.

This will enable Authors to create and download high-resolution figures. If authors have used the free BioRender service, they can use the instructions provided in the link above to download a high-resolution version suitable for publication.

The link provided should only be used for the purposes of this submission. Authors will be charged for figures created on this account if they are not related to this manuscript submission.

LANGUAGE EDITING AND SUPPORT FOR PUBLICATION: If you would like help with English language editing, or other article preparation support, Wiley Editing Services offers expert help, including English Language Editing, as well as translation, manuscript formatting, and figure formatting at www.wileyauthors.com/eeo/preparation. You can also find resources for Preparing Your Article for general guidance about writing and preparing your manuscript at www.wileyauthors.com/eeo/prepresources.

RIGOUR AND REPRODUCIBILITY: We recommend authors consult The Journal's Rigour and Reproducibility requirements to ensure that their manuscript is suitable for publication: https://physoc.onlinelibrary.wiley.com/pb-assets/hub-assets/physoc/documents/TJP-Rigour-and-Reproducibility-Requirements.pdf

REVISION CHECKLIST:

Check that your Methods section conforms to journal policy: https://jp.msubmit.net/cgi-bin/main.plex?form_type=display_requirements#methods.

Check that data presented conforms to the statistics policy: https://jp.msubmit.net/cgi-bin/main.plex?form_type=display_requirements#statistics.

Upload a full Response to Referees file. To create your 'Response to Referees': copy all the reports, including any comments from the Senior and Reviewing Editors, into a Microsoft Word, or similar, file and respond to each point, using font or background colour to distinguish comments and responses and upload as the required file type.

Please upload two versions of your manuscript text: one with all relevant changes highlighted and one clean version with no changes tracked. The manuscript file should include all tables and figure legends, but each figure/graph should be uploaded as separate, high-resolution files.

You may also upload:

- 'Potential Cover Art' for consideration as the issue's cover image
- Appropriate Supporting Information (Video, audio or data set: see https://jp.msubmit.net/cgi-bin/main.plex?form_type=display_requirements#supp).

We look forward to receiving your revised submission.

If you have any queries, please reply to this email and we will be pleased to advise.

Yours sincerely,

Bjorn Knollmann
Senior Editor
The Journal of Physiology

REQUIRED ITEMS

- Author photo and profile. First or joint first authors are asked to provide a short biography (no more than 100 words for one author or 150 words in total for joint first authors) and a portrait photograph. These should be uploaded and clearly labelled together in a Word document with the revised version of the manuscript. See Information for Authors for further details.

- Please upload separate high-quality figure files via the submission form.

- Please ensure that any tables are editable and in Word format, and wherever possible, embedded in the article file itself.

- Please ensure that the Article File you upload is a Word file.

- Please include an Abstract Figure file, as well as the Figure Legend text within the main article file. The Abstract Figure is a piece of artwork designed to give readers an immediate understanding of the research and should summarise the main conclusions. If possible, the image should be easily 'readable' from left to right or top to bottom. It should show the physiological relevance of the manuscript so readers can assess the importance and content of its findings. Abstract Figures should not merely recapitulate other figures in the manuscript. Please try to keep the diagram as simple as possible and without superfluous information that may distract from the main conclusion(s). Abstract Figures must be provided by authors no later than the revised manuscript stage and should be uploaded as a separate file during online submission labelled as File Type 'Abstract Figure'. Please also ensure that you include the figure legend in the main article file. All Abstract Figures should be created using BioRender. Authors should use The Journal's premium BioRender account to export high-resolution images. Details on how to use and access the premium account are included as part of this email.

EDITOR COMMENTS

Reviewing Editor:

The reviewers acknowledge the improvements but request that the authors better incorporate coronary autoregulation and sympathetic responsiveness into their model, and include coronary resistance in key analyses to strengthen physiological relevance. They also encourage deeper use of patient data to refine model predictions and a clearer discussion of coronary flow regulation as a potential prognostic and therapeutic target. Addressing these points will enhance the manuscript's impact and clinical significance prior to acceptance.

Senior Editor:

Both the reviewing editor and the reviewer found the study potentially interesting, but it would require major revision as outlined in the review for the work to be considered for publication.

REFEREE COMMENTS

Referee #1:

The authors present a study to assess the influence of physiological variability in fiber orientation on cardiac activation simulations. They go beyond the current state of the art by 1) using uncertainty quantification (UQ) and 2) using a full ventricle model and assessing the activation sequence spatially. While the study shows potential, the scope of the paper could hinder its impact. This could be improved to increasing the scope to include more clinically relevant and complex rhythms (e.g. sinus rhythm, VT, VF) and/or a pathological condition (e.g. ischemia, infarction). However, I understand this would be time consuming and may not be feasible for the authors. Below my comments for each section of the paper:

Introduction

-The choice of using a rule-based approach instead of an image-based one is not sufficiently justified. From a naive perspective, it would seem image-based fiber directions would be the idealdata to use in this study, given the large number of datasets available and possibility to provide the statistical distributions required for UQ. Could the authors elaborate further on the reasons for the choice and the trade-offs.

-Could the authors provide clarification on what they mean by "evaluate the activation sequence spatially", is this referring to the timing, pattern, or sequence of the activation, and how is this spatial aspect being assessed.

The objective of the study is not well stated. It would benefit from a more focused and explicit statement of what the authors aim to achieve with their work.

-The end of the introduction currently includes the conclusions of the paper. I would suggest that these would be better placed at the end of the paper, where they can synthesize the findings in context.

Methods

-There seems to be a discrepancy in how the alpha and beta angles are treated. Could the authors provide justification for why 3 distributions of the alpha angle are evaluated but only 1 for the beta angle.

-I'm unfamiliar with the concept of "global sensitivities". It would be helpful for the authors to better explain what this means, especially in the context of UQ and in this setting. Clarifying the statistical significance of this measure would be useful for readers who aren't experts in UQ methods.

-The relevance of evaluating the area and orientation of the early epicardial activation is unclear. How does this relate to real-world clinical scenarios, such as predicting arrhythmias or improving treatment planning?

Results

-The result section while comprehensive is bit repetitive making it hard to discern the key results the authors want to highlight. Summarizing key findings or moving some details to supplementary materials could improve readability. It's also important that the results are framed clearly. Currently, important information regarding the ranges of alpha angles and the different geometries tested seems to be lost or unclear in the results section.

Discussion

-Though the discussion is well-written in assessing the meaning of the presented results, I would encourage the authors to directly compare their findings with existing literature to contextualize their results and demonstrate how their study adds to or diverges from previous work. In addition, it would be interesting to know how this study impact our understanding of cardiac electrophysiology.

-The limitations section is a little too succinct. Though the different modelling choices are mentioned, it would be interesting to include some perspective of the role these choices play in relation to fibre orientation uncertainty. Especially in the case of excluding the His-Purkinje network where others have shown a drastic impact on activation sequences. Furthermore, while the impact of fibre orientation on electrophysiology has been assessed, no mention is made on the resulting mechanical contraction which is also dependent on fibre directions. Finally, it should also be noted that when using the rule-based approach there is an assumption of linear rotation of the angles from the epi to endocardial surface. This may not be the correct model for the rotation.

END OF COMMENTS

Response to Reviews from Submission JP-RP-2025-287746: Uncertainty Quantification via Polynomial Chaos Expansion of Myocardial Fiber Orientation and Cardiac Activation Patterns

Lindsay C. R. Tanner[1,2,3,*], Anna Busatto[1,2,3], Jake A. Bergquist[1,2,3], Wilson W. Good[1,2,3], Brian Zenger[4], Gernot Plank[5], Akil Narayan[1,6], Karli Gillette[1,3,5], Rob S. MacLeod[1,2,3]

[1] Scientific Computing and Imaging Institute, University of Utah, Salt Lake City, Utah, United States of America
[2] Nora Eccles Cardiovascular Research and Training Institute, University of Utah, Salt Lake City, Utah, United States of America
[3] Department of Biomedical Engineering, University of Utah, Salt Lake City, Utah, United States of America
[4] Washington University School of Medicine in Saint Louis, Saint Louis Missouri, United States of America
[5] Division of Biophysics and Medical Physics, Gottfried Schatz Research Center, Medical University of Graz, Graz, Austria
[6] Department of Mathematics, University of Utah, Salt Lake City, Utah, United States of America

* Corresponding Author: lindsay.c.r.tanner@utah.edu

June 7, 2025

We would like to sincerely thank the reviewers for their time, thoughtful comments, and valuable suggestions regarding our manuscript. We greatly appreciate the opportunity to revise our work based on the feedback. In response to these comments, we have made substantial revisions to the manuscript. To aid in the review process, we have color-coded the changes within the revised manuscript as follows:

1. Red for revisions addressing **Reviewer 1**.
2. Blue for revisions addressing **Reviewer 2**.
3. Green for revisions addressing **multiple reviewers**.

We have provided detailed, point-by-point responses to each reviewer's comment below. We hope that the changes meet their expectations and improve the overall quality and clarity of the manuscript.

1 Reviewer 1

The authors present a study to assess the influence of physiological variability in fiber orientation on cardiac activation simulations. They go beyond the current state of the art by 1) using uncertainty quantification (UQ) and 2) using a full ventricle model and assessing the activation sequence spatially. While the study shows potential, the scope of the paper could hinder its impact. This could be improved to increasing the scope to include more clinically relevant and complex rhythms (e.g. sinus rhythm, VT, VF) and/or a pathological condition (e.g. ischemia, infarction). However, I understand this would be time consuming and may not be feasible for the authors. Below my comments for each section of the paper:

We appreciate the reviewer's comment and agree that incorporating more complex rhythms or pathological conditions would enhance the clinical impact of this work. These are precisely our plans for future studies, however, there are many obstacles to these next steps. Realistic simulation of cardiac activation in whole-heart models is a topic of active research and we are actively pursuing this goal.

Moreover, there are no previous studies that address the impact of uncertainty in fiber structure at the level of precision and sophistication we have completed. The field is remarkably immature in addressing these concerns, and our goal was to illustrate that a UQ analysis is both feasible and insightful. Our results suggest that progress in simulating cardiac activity must proceed in close recognition of the importance of uncertainty and its impact on the output of these simulations. The impact of our contribution lies in the evidence that UQ is both necessary and feasible, just as it is embarrassingly absent from most studies.

Our primary objective was to evaluate the effect of physiological variability in fiber orientation in a controlled setting using uncertainty quantification. Starting with ectopic activation in a healthy ventricular model provides a simplified substrate in which it is possible to systematically assess how fiber orientation influences activation patterns. This foundational insight is critical before extending the methodology to more complex cases. Furthermore, the methods we developed—particularly the UQ framework and spatial analysis tools—are generalizable and can be applied directly to future studies involving sinus rhythm, arrhythmias (*e.g.,* VT, VF), or pathological remodeling (*e.g.,* post-infarct scar or ischemia).

Introduction

1. *The choice of using a rule-based approach instead of an image-based one is not sufficiently justified. From a naive perspective, it would seem image-based fiber directions would be the ideal data to use in this study, given the large number of datasets available and possibility to provide the statistical distributions required for UQ. Could the authors elaborate further on the reasons for the choice and the trade-offs.*

   We have added clarification in both the Introduction (lines X-X) and Discussion (lines

X-X) to address this point. While image-based data may seem ideal, in practice, they remain limited: high-quality DT-MRI is rarely available, especially in a form suitable for patient-specific or large-scale UQ studies. Diffusion-tensor MRI in a beating heart is in its earliest phase of research and results to date have been discouraging. Rule-based methods, by contrast, allow systematic, parameterized control of fiber orientation across many models, something not feasible with existing imaging datasets. This consistency and scalability made rule-based modeling the only practical choice for our study.

2. *Could the authors provide clarification on what they mean by "evaluate the activation sequence spatially", is this referring to the timing, pattern, or sequence of the activation, and how is this spatial aspect being assessed.*

   By "evaluate the activation sequence spatially," we mean assessing the timing, pattern, and progression of activation through the heart volume and across the heart surface. The eikonal model provides activation times at every three-dimensional location, providing both spatial and temporal information. Our analysis was limited to the epicardial surface for several pragmatic reasons. Visualizing a time-dependent behavior in three dimensions is intrinsically challenging, especially within the constraints of journals. Measurements, whether clinical or in experiments, are also typically limited to the heart surfaces, endocardial or epicardial. Combining the timing data with these spatial patterns allows for useful characterization of the sequence of activation propagation.

3. *The objective of the study is not well stated. It would benefit from a more focused and explicit statement of what the authors aim to achieve with their work.*

   We have added a clear and explicit goal statement to the Introduction on lines X-X to better focus and clarify the objective of the study.

4. *The end of the introduction currently includes the conclusions of the paper. I would suggest that these would be better placed at the end of the paper, where they can synthesize the findings in context.*

   This is a stylistic consideration we are happy to work with. We have removed the concluding statements from the end of the introduction, as they were synthesized in the discussion section.

Methods

1. *There seems to be a discrepancy in how the alpha and beta angles are treated. Could the authors provide justification for why 3 distributions of the alpha angle are evaluated but only 1 for the beta angle.*

   We evaluated only one distribution for the beta angle because we found, in preliminary studies, that its variability had only a negligible effect on activation times compared to the alpha angle. Even when testing relatively large ranges for the beta angle, its impact on activation was minimal, whereas variability in the alpha angle showed a clear influence. Therefore, we focused on multiple alpha angle distributions.

2. *I'm unfamiliar with the concept of "global sensitivities". It would be helpful for the authors to better explain what this means, especially in the context of UQ and in this setting. Clarifying the statistical significance of this measure would be useful for readers who aren't experts in UQ methods.*

   We added a clearer explanation of global sensitivities on lines X–X in the Methods section. Specifically, we now define them as measures of how much each input parameter contributes to output variability not just locally, but across its entire range. We have also added an explanation of how these are variance-based indices showed which fiber orientation angles affected activation times.

3. *The relevance of evaluating the area and orientation of the early epicardial activation is unclear. How does this relate to real-world clinical scenarios, such as predicting arrhythmias or improving treatment planning?*

   While these metrics may not directly translate to clinical applications, this does not diminish their relevance for our study's goals. The clinical evaluation of early activation is relevant when the goal is to identify and treat frequent PVC's. From the mechanistic perspective, previous research by Bruno Taccardi has demonstrated in detailed animal experiments that the area and orientation of the early epicardial activation isochrone are sensitive to fiber orientation and pacing depth. We embraced these established metrics and sought to assess whether and how they vary with fiber orientation variability. This approach allows us to better understand the mechanistic influence of fiber architecture on activation patterns, which is a critical step before such findings can be connected to clinical scenarios like PVC localization, arrhythmia prediction, or treatment planning. We have added this clarification on lines X-X of the manuscript.

Results

1. *The result section while comprehensive is bit repetitive making it hard to discern the key results the authors want to highlight. Summarizing key findings or moving some details to supplementary materials could improve readability. It's also important that the results are framed clearly. Currently, important information regarding the ranges of alpha angles and the different geometries tested seems to be lost or unclear in the results section.*

   We appreciate the suggestion to improve clarity and focus in the Results section. The results included are already a condensation of the studies we conducted and we consider all of them important to fully support our key findings. However, we have restructured and trimmed the Results to improve readability and highlighted the main points more clearly by adding additional subsections and organizing content in a way we hope is more logical.

   Regarding supplementary materials, the journal does not allow them. For that, there are no restrictions on manuscript length or number of figures and tables, as long as manuscripts areas clear and concise as possible, with figures and tables limited to what is necessary to illustrate the hypothesis and support conclusions. Therefore, all relevant results have been retained within the main text with improved structure to enhance clarity.

Discussion

1. *Though the discussion is well-written in assessing the meaning of the presented results, I would encourage the authors to directly compare their findings with existing literature to contextualize their results and demonstrate how their study adds to or diverges from previous work. In addition, it would be interesting to know how this study impacts our understanding of cardiac electrophysiology.*

   While our original discussion included some references to prior experimental and computational work, we have added a new paragraph to more explicitly situate our results within the broader landscape of uncertainty quantification in cardiac electrophysiological modeling. This addition highlights related efforts in the field and reinforces the relevance of studying fiber orientation uncertainty within the wider context of electrophysiological modeling.

2. *The limitations section is a little too succinct. Though the different modelling choices are mentioned, it would be interesting to include some perspective of the role these choices play in relation to fibre orientation uncertainty. Especially in the case of excluding the His-Purkinje network where others have shown a drastic impact on activation sequences. Furthermore, while the impact of fibre orientation on electrophysiology has been assessed, no mention is made on the resulting mechanical contraction which is also dependent on fibre directions. Finally, it should also be noted that when using the rule-based approach there is an assumption of linear rotation of the angles from the epi to endocardial surface. This may not be the correct model for the rotation.*

   As suggested, we have revised the discussion section to provide a more detailed and explicit assessment of the limitations of our study. Specifically, we have expanded our discussion of how key modeling choices, such as excluding the His-Purkinje network, using rule-based fiber orientation, and employing the eikonal approximation, relate to our uncertainty quantification analysis of fiber orientation. We hope these additions better contextualize our findings and clarify the scope and implications of our modeling framework. Please see lines X–X, and X-X for these revisions.

2 Reviewer 2

Tanner et al. perform a systematic and comprehensive uncertainty quantification study on the effects of ventricular fibers on activation patterns. The study is well executed and presented. I have the following questions or points of clarification:

1. *What does the following mean: "The hearts exhibited volumetric similarity within 3.8%"?*

   To clarify, we have replaced the percentage expression with the actual volumes of the hearts to more clearly demonstrate their similarity. Please see Line X for this change.

2. *Why did you use polynomial chaos expansion approach and eikonal and not GPE?*

We chose the polynomial chaos expansion method because it provides a computationally efficient way to quantify uncertainty in our model outputs with a relatively low number of simulation runs. PCE is particularly well suited for problems where the input uncertainties can be represented with probability distributions, allowing us to approximate the output response surface with orthogonal polynomials, which yields accurate statistical moments and sensitivity indices.

On the other hand, while Gaussian Process Emulators (GPEs) are powerful for surrogate modeling, especially with smaller datasets or highly nonlinear responses, they can be computationally expensive to train. Given the dimensionality of our input space and the nature of our problem, PCE combined with the eikonal approximation provided a more straightforward and scalable framework for our uncertainty quantification goals. That said, GPEs remain a promising alternative, and future studies are needed to explore their integration for comparison.

3. *"For the entire myocardium, longitudinal conduction velocity was set to 100 cm/s (Good et al., 2018), and the transverse and sheet conduction velocities were defined as 2/3 and 1/3 of the longitudinal value, respectively (Taggart et al., 2000)." Given the conductivity values - how sensitive are the predictions to these values and the anisotropy ratio?*

   We completely agree that conduction velocity and anisotropy ratios can influence simulation outcomes. While the research we presented did not systematically vary these parameters to assess sensitivity, we are finalizing a follow-up study specifically designed to investigate how variability in conduction velocities influences eikonal-based cardiac simulations.

4. *Effects of noise in fibers, or effects of border zone/ fibrosis / infarct on fibers?*

   While we have not explicitly quantified noise or variability in the rule-based fiber orientations used in this study, we have included a discussion (see Line X & Y) addressing how ill-defined or noisy fiber regions could potentially influence our uncertainty quantification (UQ) results.

   Regarding the effects of border zones, fibrosis, or infarction on fiber orientation and conduction, these complex tissue heterogeneities introduce conduction abnormalities that are not captured in the current model. A dedicated study incorporating these pathological conduction changes would be necessary to rigorously evaluate the impact of fiber orientation and subsequent simulation outcomes. At present, we can only hypothesize about these effects, as empirical data or detailed modeling of such scenarios are topics of ongoing research.

5. *Expected effects on mechanics?*

   We agree that fiber orientation plays a critical role in cardiac mechanics. However, the current study is focused exclusively on the electrical properties and their uncertainty. To investigate the effects on cardiac mechanics, a new analysis incorporating a mechanical or electro-mechanical modeling framework would be required. For example, it is naive to assume that mechanics follow inevitably and strictly from the electrophysiology; recent

studies suggest that mechano-electrophysiological feedback occurs and must be incorporated in realistic mechamnics models. This topic represents an important direction for future work beyond the scope of the present study.

6. *Any difference with monodomain instead of eikonal?*

   As stated in the discussion, previous studies show that eikonal and bidomain models produce comparable results (Neic et al. doi: 10.1016/j.jcp.2017.06.020) under the conditions we applied. We can only assume that the monodomain model, being intermediate in complexity compared to the bidomain approach, would likely yield similar qualitative outcomes. A further justification of the eikonal approach is our focus on activation variability due to fiber orientation, which is well and efficiently captured by the eikonal approach.

7. *"Since the sample sets also include some degree of random selection," - please explain this point.*

   We have clarified this point on Line X. While the sample sets are generated using a randomized procedure, the method is designed to reduce variability in accuracy by guiding the random selection through a least-squares-based strategy. This method ensures both randomness and robustness in the sampling process.

8. *Figure 2: For the global sensitivity contributions, what are the metrics? I suggest using a different colour map if not Act Time.*

   The global sensitivity contributions shown in Figure 2 are based on activation time as the output metric. While we acknowledge that color-map design is an active area of research within the scientific visualization community, we opted to use a consistent color map across figures to facilitate interpretation and comparison. Maintaining a uniform visual scheme helps highlight spatial patterns and regions of interest.

9. *Results 6.2 Beta angle - any table?*

   We have now included a table summarizing the standard deviations associated with variability in the beta fiber angle across all stimulus sites (see Table 4).

10. *Any rate dependence expected - would repolarisation vary and re-entry mechanisms?*

    Investigating rate dependence, repolarization dynamics, and re-entry mechanisms are all incredibly intriguing questions that would require a more detailed analysis of depolarization-repolarization coupling and arrhythmia pathways—and a whole different modeling framework to capture the behavior. Our current study focuses solely on activation (depolarization) patterns under controlled conditions and does not include the necessary modeling components to directly assess re-entry or repolarization behavior. However, our findings may provide useful insights that could inform future studies exploring these more complex phenomena. Our central idea is that, just as any experimental study requires statistical analysis, any simulation study must include uncertainty quantification.

3 Other

1. *Author photo and profile. First or joint first authors are asked to provide a short biography (no more than 100 words for one author or 150 words in total for joint first authors) and a portrait photograph. These should be uploaded and clearly labelled together in a Word document with the revised version of the manuscript. See Information for Authors for further details.*

   We have now included the author photo and short biography within the revised manuscript using LaTeX, as per the journal's formatting guidelines.

2. *Please upload separate high-quality figure files via the submission form.*

   We have uploaded all figures as separate high-quality files via the submission form, as requested.

3. *Please ensure that any tables are editable and in Word format, and wherever possible, embedded in the article file itself.*

   Our manuscript was prepared in LaTeX, and all tables are embedded directly within the article file and are also included in pdf format.

4. *Please ensure that the Article File you upload is a Word file.*

   Our manuscript was prepared in LaTeX and is currently not in Word format. However, while we can convert and provide a Word version if required, we note that LaTeX is a format used widely in scientific publications, many of which provide journal-specific templates.

5. *Please include an Abstract Figure file, as well as the Figure Legend text within the main article file. The Abstract Figure is a piece of artwork designed to give readers an immediate understanding of the research and should summarise the main conclusions. If possible, the image should be easily 'readable' from left to right or top to bottom. It should show the physiological relevance of the manuscript so readers can assess the importance and content of its findings. Abstract Figures should not merely recapitulate other figures in the manuscript. Please try to keep the diagram as simple as possible and without superfluous information that may distract from the main conclusion(s). Abstract Figures must be provided by authors no later than the revised manuscript stage and should be uploaded as a separate file during online submission labelled as File Type 'Abstract Figure'. Please also ensure that you include the figure legend in the main article file. All Abstract Figures should be created using BioRender. Authors should use The Journal's premium BioRender account to export high-resolution images. Details on how to use and access the premium account are included as part of this email.*

   We previously included an abstract figure (see Figure 1), which was formatted using Adobe Illustrator. However, the underlying data-driven visualizations, specifically, the mapping of activation data onto three-dimensional anatomical surfaces, were generated using specialized software not supported by BioRender. Illustrator is a highly regarded professional tool for creating figures and was used solely for formatting and layout, as it allowed us to align and annotate the images for clarity. The current version was exported at high resolution and adheres to the journal's quality standards.

Dear Mrs Tanner,

Re: JP-RP-2025-287746R1 "Uncertainty Quantification via Polynomial Chaos Expansion of Myocardial Fiber Orientation and Cardiac Activation Patterns" by Lindsay C.R. Tanner, Anna Busatto, Jake A Bergquist, Wilson W Good, Brian Zenger, Gernot Plank, Akil Narayan, Karli Gillette, and Rob S. MacLeod

We are pleased to tell you that your paper has been accepted for publication in The Journal of Physiology.

The last Word (or similar) version of the manuscript provided will be used by the Production Editor to prepare your proof. When this is ready you will receive an email containing a link to Wiley's Online Proofing System. The proof should be thoroughly checked and corrected as promptly as possible.

Authors should note that it is too late at this point to offer corrections prior to proofreading. Major corrections at proof stage, such as changes to figures, will be referred to the Editors for approval before they can be incorporated. Only minor changes, such as to style and consistency, should be made at proof stage. Changes that need to be made after proof stage will usually require a formal correction notice.

All queries at proof stage should be sent to: TJP@wiley.com.

Following copyediting and typesetting the accepted version will be published online.

Yours sincerely,

Bjorn Knollmann
Senior Editor
The Journal of Physiology

IMPORTANT POINTS TO NOTE FOLLOWING YOUR PAPER'S ACCEPTANCE:

If you would like to receive our 'Research Roundup', a monthly newsletter highlighting the cutting-edge research published in The Physiological Society's family of journals (The Journal of Physiology, Experimental Physiology, Physiological Reports, The Journal of Nutritional Physiology and The Journal of Precision Medicine: Health and Disease), please click this link, fill in your name and email address and select 'Research Roundup':
https://www.physoc.org/journals-and-media/membernews

• TRANSPARENT PEER REVIEW POLICY: To improve the transparency of its peer review process, The Journal of Physiology publishes online as supporting information the peer review history of all articles accepted for publication. Readers will have access to decision letters, including Editors' comments and referee reports, for each version of the manuscript as well as any author responses to peer review comments. Referees can decide whether or not they wish to be named on the peer review history document.

• You can help your research get the attention it deserves! Check out Wiley's free Promotion Guide for best-practice recommendations for promoting your work at: www.wileyauthors.com/eeo/guide. You can learn more about Wiley Editing Services which offers professional video, design, and writing services to create shareable video abstracts, infographics, conference posters, lay summaries, and research news stories for your research at: www.wileyauthors.com/eeo/promotion.

• IMPORTANT NOTICE ABOUT OPEN ACCESS: To assist authors whose funding agencies mandate public access to published research findings sooner than 12 months after publication, The Journal of Physiology allows authors to pay an Open Access (OA) fee to have their papers made freely available immediately on publication.

The Corresponding Author will receive an email from Wiley with details on how to register or log-in to Wiley Authors Services where you will be able to place an order.

• You can check if your funder or institution has a Wiley Open Access Account here: https://authorservices.wiley.com/author-resources/Journal-Authors/licensing-and-open-access/open-access/author-compliance-tool.html.

EDITOR COMMENTS

Reviewing Editor:

Thank you for addressing all comments and, the manuscript seems ready for acceptance.

Senior Editor:

The MS is now acceptable.

REFEREE COMMENTS

Referee #1:

The authors have sufficiently addressed my comments.

Referee #2:

Thank you for addressing all of my comments - congrats on a brilliant study!
